# The quality of life and work productivity are affected by the presence of nausea/vomiting in patients taking iron preparations for heavy menstrual bleeding or anemia: a population-based cross-sectional survey in Japan

**DOI:** 10.1186/s12905-024-03104-0

**Published:** 2024-05-21

**Authors:** Kyoko Ito, Yuko Mitobe, Ryo Inoue, Mikio Momoeda

**Affiliations:** 1 Medical Affairs Department, Torii Pharmaceutical Co., Ltd., 3-4-1 Nihonbashi-Honcho, Chuo-Ku, Tokyo, 103-8439 Japan; 2 Aiiku Maternal and Child Health Center, Aiiku Hospital, 1-16-10 Shibaura, Minato-Ku, Tokyo, 105-8321 Japan

**Keywords:** Iron preparations, Nausea, Vomiting, QOL, WPAI, Work productivity

## Abstract

**Background:**

Patients with iron deficiency anemia are treated with iron preparations, but gastrointestinal symptoms such as nausea and vomiting occur frequently. These symptoms may negatively affect the quality of life and work productivity in patients with iron deficiency anemia. This study assessed the impact of nausea and vomiting on the quality of life and work productivity of patients taking iron preparations for heavy menstrual bleeding or anemia.

**Methods:**

An online survey was conducted among patients taking iron preparations for heavy menstrual bleeding or anemia. Demographic data and information about medication use and the health condition were collected. The patients were asked to answer the 5-level EQ-5D version, and work productivity and activity impairment questionnaires. The outcomes were reported by patients in the presences of nausea, vomiting, and nausea or vomiting. The association with the 5-level EQ-5D version utility score for the severity and frequency of the symptoms were also assessed.

**Results:**

A total of 385 patients were enrolled, and 96 were patients with nausea or vomiting, of which 94 were with nausea and 27 were with vomiting. The 5-level EQ-5D version utility scores for the patients with nausea, vomiting, and nausea or vomiting were significantly lower than those of the patients without these symptoms (*p* < 0.001 for each). The 5-level EQ-5D version utility score was correlated with the severity of nausea and the frequency of vomiting per day (*p* < 0.001 for each). As for the work productivity and activity impairment, the presenteeism, the overall work impairment, and the activity impairment of the patients with nausea, vomiting, and nausea or vomiting were significantly higher than those without these symptoms (*p* < 0.001 for each). The absenteeism was slightly higher trend was observed, but not significant.

**Conclusion:**

Patients taking iron preparations who have nausea or vomiting experience a significant burden in terms of poorer quality of life and higher work productivity impairment.

**Trial registration:**

UMIN000045700 (http://www.umin.ac.jp/ctr/). Registered on October 11, 2021.

## Background

Anemia is an important global health problem. Worldwide, 29.6% of non-pregnant women and 36.5% of pregnant women were affected by anemia in 2019 [1]. As reported by the Japanese Ministry of Health, Labour and Welfare (MHLW) in 2018, anemia, defined as Hb < 12 g/dL, is observed in 13.9% of Japanese adult women and 20.2% and 22.7% of women in their 30s and 40s, respectively in 2018, suggesting higher prevalence among those in their 30s and 40s [2].

Although anemia has various correlates based on its underlying pathophysiology, iron deficiency, a common cause of anemia in women, results from inadequate dietary iron intake or absorption, increased iron demand during pregnancy, and increased menstrual iron loss [3]. The clinical presentations and complications of anemia differ according to the type of anemia and its level of severity. The consequences of iron deficiency can result in a wide variety of adverse outcomes including impaired thermoregulation, immune dysfunction, gastrointestinal disturbances, and neurocognitive impairment [4]. Iron deficiency, even in the absence of anemia, can also have negative effects, including clinical signs and symptoms such as fatigue, impaired physical performance, and decreased work productivity and social activities [5].

In patients with the symptoms of iron deficiency anemia, treatment with iron preparations, as well as the treatment of causative diseases and improvement of lifestyle, is performed. However, gastrointestinal issues with oral iron preparations significantly increased risk of gastrointestinal symptoms [6]. Gastrointestinal adverse effects, such as nausea and vomiting, appear in up to 30% of patients taking iron preparations, which decreases patients’ adherence to appropriate treatment [7–9]. The adverse effects of iron preparations may affect the quality of life (QOL) and work productivity of patients with the symptoms of iron deficiency anemia.

To examine the impacts of the adverse effects on the QOL in patients taking iron preparations, we collected 5-level EQ-5D version (EQ-5D-5L) utility scores and other patient reported outcomes (PROs) from a web-based survey in 385 patients taking iron preparations for heavy menstrual bleeding (HMB) or anemia to construct a formula for estimating the impacts of nausea or vomiting and other PROs on the EQ-5D-5L utility scores. As a result, the reductions in the EQ-5D-5L utility scores were estimated to be -0.117 for either nausea or vomiting and -0.081 for the symptoms of anemia [10].

To the best of our knowledge, the impacts of nausea and vomiting caused by iron preparation intake on productivity have not been reported, although several studies have reported the impacts of nausea and vomiting on productivity. As reported by Piwko et al., nausea and vomiting are significantly associated with productivity in pregnant women, and, as shown by the self-reported severities of symptoms (mild, moderate, and severe), losses are greater for moderate than mild symptoms and for severe than moderate symptoms [11]. Gajria et al. conducted a study in migraineurs, revealing that activity impairment and work productivity loss costs were significantly greater in patients with nausea and vomiting than in those without such symptoms [12]. A Japanese study reported the impacts of the adverse effects, nausea and vomiting caused by outpatient chemotherapy in breast cancer patients, on QOL and work productivity [13, 14]. Thus, nausea and vomiting, caused by iron preparation intake, may have impacts on work productivity, as well as QOL.

We previously reported the impacts of any of nausea and vomiting caused by iron preparation intake on QOL [10]. However, no report has been published on the differences in the impacts of any of nausea and vomiting on QOL, the relationship between the self-reported severities of symptoms (nausea and vomiting) and QOL, and the work productivity impairment due to nausea and vomiting.

The objective of this study was to assess, via a web-based survey, the individual impact of nausea and vomiting on QOL and work productivity impairment in patients taking iron preparations for HMB or anemia.

## Methods

### Study design

We conducted an online web-based survey between 22 and 25 October 2021 among patients taking iron preparations for HMB or anemia. The survey was a self-administered, self-reported, internet-based questionnaire for women 20 years of age or older. The respondents were recruited through a patient panel of Japanese residents who agree to participate regularly in online surveys (INTAGE Healthcare Inc., Tokyo, Japan) [10].

The patients taking iron preparations were defined as those who have taken iron preparations (intravenous, oral, oral supplements) at least within the past 3 months. Patients were included who met the following 4 criteria; (i) have HMB or anemia, (ii) have taken iron preparations at least within the past 3 months, (iii) 20 years of age or older at the time of the survey and (iv) have access to an internet-capable device (computer, smartphone, tablet) and are able to operate it, or have the cooperation of someone who can operate it. Patients who did not agree to participate in this study were excluded. The protocol of the study was approved by the ethics review committee, “Non-Profit Organization MINS Institutional Review Board” on September 16, 2021, and clinical trial registration was made on October 11, 2021 (UMIN000045700). Before this study was conducted, electronic consent was obtained from all subjects. Additional details, including sample size determination, were provided previously [10].

### Measures

The questionnaire included questions on demographics [sex, age, and primary disease (endometriosis, uterine myoma, adenomyosis uteri, endometrial polyps, dysmenorrhea, premenstrual syndrome (PMS), HMB, others)], medication use [low-dose oral contraceptives, iron preparations (intravenous, oral, oral supplements), estrogen preparation, others], whether during menstruation period, presence of current symptoms (nausea, vomiting, PMS, menstrual pain, menstrual symptoms, anemia), duration and severity of nausea, duration and frequency of vomiting, employee status, EQ-5D-5L utility score, and work productivity and activity impairment (WPAI).

The severity of nausea was assessed by a numerical rating scale (NRS) ranging from 0 to 10. A score of 0 indicates no nausea while 10 represents the worst nausea possible.

The EQ-5D-5L questionnaire was developed by EuroQol Group as a standardized non-disease specific instrument to describe and value health-related QOL which includes five dimensions of health states (mobility, self-care, usual activities, pain/discomfort, and anxiety/depression) and a visual analog scale (EQ-5D VAS) ranging from 0 to 100. A score of 0 indicates the worst health a patient can imagine while 100 represents the best health a patient can imagine [15]. The EQ-5D-5L utility scores were derived using the Japanese EQ-5D-5L tariffs developed by Shiroiwa et al. [16].

WPAI is a six–item validated questionnaire that measures the metrics of problems with work during the past 7 days. WPAI evaluates the percent impairment while working (i.e., presenteeism), percent work time missed (i.e., absenteeism), percent overall work impairment (OWI) (i.e., combination of absenteeism and presenteeism), and percent daily activity impairment (AI). The score shows the percentage of hours missed due to problems and the degree health affected productivity. The greater percentage of absenteeism indicates the greater burden, while the greater percentage of presenteeism indicates more time spent at work [17].

### Statistical analyses

All outcomes were reported using counts and percentages for categorical variables and means and standard deviations (SD) for continuous variables and compared by patient in the presence of nausea, the presence of vomiting, and the presence of nausea/vomiting. If at least one of the responses of “nausea” or “vomiting” was selected as a current symptom, it was defined as “with nausea/vomiting”. Significant differences between groups were analyzed using X^2^ tests for categorical variables and the T test for continuous variables.

Patients taking iron preparations for HMB or anemia were included in the study, however, some patients without symptoms of anemia were included. Therefore, a sensitivity analysis was performed only on patients with symptoms of anemia. For the sensitivity analysis, the EQ-5D-5L utility score and the WPAI scores were assessed only in patients presenting with the symptoms of anemia.

Furthermore, in patients presenting with nausea/vomiting, the association with the EQ-5D-5L utility score for each of the EQ VAS score, duration and severity of nausea, duration of vomiting, and frequency of vomiting was assessed using Pearson correlation coefficients.

All statistical analyses were conducted in SAS version 9.4 (SAS Institute Inc, Cary, NC, USA) or Stata version 15.0 (Stata Corp LP, College Station, TX, USA). No correction for multiple testing was conducted as no formal hypothesis testing was planned for this study. P values were provided as a measure of group differences and *p* values < 0.05 were considered to be statistically significant.

## Results

### Characteristics of the study participants

**Fig. 1:**
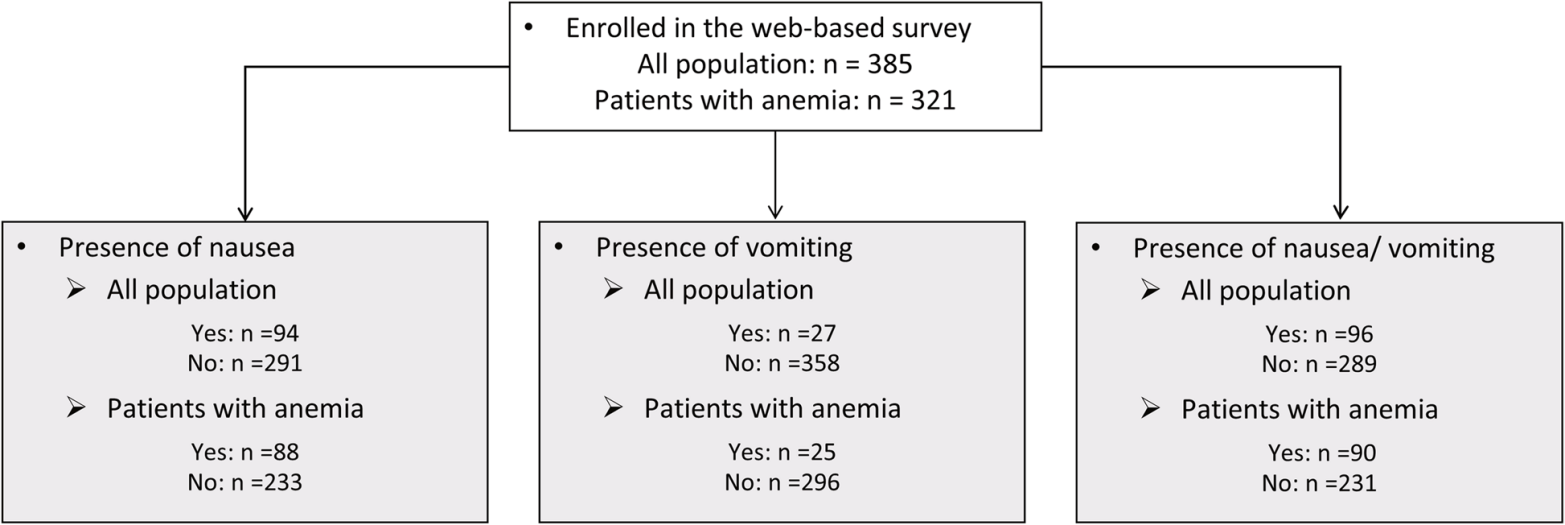
Flowchart of analysis population

The patient characteristics for all the patients and patients with the presence of nausea or vomiting are shown in Table 1. A total of 385 patients were enrolled in the web-based survey. Of the 385 patients, 96 were patients with nausea/vomiting, of which 94 were with nausea and 27 were with vomiting (Fig. 1), and 25 out of 27 patients were with both nausea and vomiting (data not shown). All the patients were female with a mean age of 41.6 ± 7.7 years (Mean ± SD).

**Table 1:** Characteristics of study participants

	All	Presence of nausea			Presence of vomiting			Presence of nausea/vomiting		
	Total *N* = 385	Yes *N* = 94	No *N* = 291	*p*-value	Yes *N* = 27	No *N* = 358	*p*-value	Yes *N* = 96	No *N* = 289	*p*-value
Age	41.6 (7.7)	39.6 (8.0)	42.3 (7.5)	0.004	38.5 (6.8)	41.9 (7.8)	0.030	39.6 (8.0)	42.3 (7.5)	0.002
Primary disease										
All^a^	174 (45.2%)	38 (40.4%)	136 (46.7%)	0.290	14 (51.9%)	160 (44.7%)	0.470	39 (40.6%)	135 (46.7%)	0.300
Endometriosis	56 (14.5%)	18 (19.1%)	38 (13.1%)	0.150	11 (40.7%)	45 (12.6%)	< 0.001	19 (19.8%)	37 (12.8%)	0.092
Uterine myoma	130 (33.8%)	27 (28.7%)	103 (35.4%)	0.230	10 (37.0%)	120 (33.5%)	0.710	27 (28.1%)	103 (35.6%)	0.180
Adenomyosis uteri	35 ( 9.1%)	9 ( 9.6%)	26 ( 8.9%)	0.850	5 (18.5%)	30 ( 8.4%)	0.077	9 ( 9.4%)	26 ( 9.0%)	0.910
Endometrial polyp	6 ( 1.6%)	5 ( 5.3%)	1 ( 0.3%)	< 0.001	3 (11.1%)	3 ( 0.8%)	< 0.001	5 ( 5.2%)	1 ( 0.3%)	< 0.001
Dysmenorrhea	88 (22.9%)	34 (36.2%)	54 (18.6%)	< 0.001	15 (55.6%)	73 (20.4%)	< 0.001	36 (37.5%)	52 (18.0%)	< 0.001
Premenstrual syndrome	63 (16.4%)	29 (30.9%)	34 (11.7%)	< 0.001	14 (51.9%)	49 (13.7%)	< 0.001	31 (32.3%)	32 (11.1%)	< 0.001
HMB	90 (23.4%)	24 (25.5%)	66 (22.7%)	0.570	10 (37.0%)	80 (22.3%)	0.082	24 (25.0%)	66 (22.8%)	0.660
Others	27 ( 7.0%)	2 ( 2.1%)	25 ( 8.6%)	0.033	0 ( 0.0%)	27 ( 7.5%)	0.140	2 ( 2.1%)	25 ( 8.7%)	0.029
Medication use										
Low dose oral contraceptives’	64 (16.6%)	21 (22.3%)	43 (14.8%)	0.087	7 (25.9%)	57 (15.9%)	0.180	22 (22.9%)	42 (14.5%)	0.056
Iron preparation (intravenous)	39 (10.1%)	15 (16.0%)	24 ( 8.2%)	0.031	8 (29.6%)	31 ( 8.7%)	< 0.001	15 (15.6%)	24 ( 8.3%)	0.039
Iron preparation (oral)	266 (69.1%)	58 (61.7%)	208 (71.5%)	0.075	17 (63.0%)	249 (69.6%)	0.470	59 (61.5%)	207 (71.6%)	0.062
Iron preparation (oral supplement)	146 (37.9%)	46 (48.9%)	100 (34.4%)	0.011	12 (44.4%)	134 (37.4%)	0.470	47 (49.0%)	99 (34.3%)	0.010
Estrogen preparation	71 (18.4%)	24 (25.5%)	47 (16.2%)	0.041	9 (33.3%)	62 (17.3%)	0.039	24 (25.0%)	47 (16.3%)	0.056
Others	52 (13.5%)	13 (13.8%)	39 (13.4%)	0.920	5 (18.5%)	47 (13.1%)	0.430	13 (13.5%)	39 (13.5%)	0.990
During menstrual period	114 (29.6%)	37 (39.4%)	77 (26.5%)	0.017	16 (59.3%)	98 (27.4%)	< 0.001	39 (40.6%)	75 (26.0%)	0.006
Current symptoms										
Menstrual pain^b^	96 (84.2%)	36 (97%)	60 (78%)	0.008	16 (100%)	80 (82%)	0.062	38 (97%)	58 (77%)	0.005
Premenstrual syndrome	100 (26.0%)	30 (31.9%)	70 (24.1%)	0.130	10 (37.0%)	90 (25.1%)	0.170	30 (31.3%)	70 (24.2%)	0.170
Menstrual symptoms	214 (55.6%)	67 (71.3%)	147 (50.5%)	< 0.001	26 (96.3%)	188 (52.5%)	< 0.001	69 (71.9%)	145 (50.2%)	< 0.001
Anemia	321 (83.4%)	88 (93.6%)	233 (80.1%)	0.002	25 (92.6%)	296 (82.7%)	0.180	90 (93.8%)	231 (79.9%)	0.002
Nausea	94 (24.4%)	94 (100.0%)	0 ( 0.0%)	< 0.001	25 (92.6%)	69 (19.3%)	< 0.001	94 (97.9%)	0 ( 0.0%)	< 0.001
Vomiting	27 ( 7.0%)	25 (26.6%)	2 ( 0.7%)	< 0.001	27 (100.0%)	0 ( 0.0%)	< 0.001	27 (28.1%)	0 ( 0.0%)	< 0.001
Nausea duration^c^	17.0 (101.7)	17.0 (101.7)	-	-	45.4 (196.9)	6.7 (8.2)	0.100	17.0 (101.7)	-	-
Nausea severity^c^	4.5 (2.2)	4.5 (2.2)	-	-	5.2 (2.8)	4.3 (1.9)	0.080	4.5 (2.2)	-	-
Vomiting duration^d^	40.4 (189.8)	43.5 (197.2)	1.5 (0.7)	0.770	40.4 (189.8)	-	-	40.4 (189.8)	-	-
Vomiting frequency (times/day)^d^	2.0 (2.2)	2.1 (2.3)	1.5 (0.7)	0.730	2.0 (2.2)	-	-	2.0 (2.2)	-	-
Employee status										
Full time employee	122 (31.7%)	22 (23.4%)	100 (34.4%)	0.047	11 (40.7%)	111 (31.0%)	0.290	24 (25.0%)	98 (33.9%)	0.100
Company executive	1 (0.3%)	0 ( 0.0%)	1 ( 0.3%)	0.570	0 ( 0.0%)	1 ( 0.3%)	0.780	0 ( 0.0%)	1 ( 0.3%)	0.560
Self-employee	7 (1.8%)	1 ( 1.1%)	6 ( 2.1%)	0.530	0 ( 0.0%)	7 ( 2.0%)	0.460	1 ( 1.0%)	6 ( 2.1%)	0.510
Part time job	143 (37.1%)	44 (46.8%)	99 (34.0%)	0.026	13 (48.1%)	130 (36.3%)	0.220	44 (45.8%)	99 (34.3%)	0.042
Homemaker	78 (20.3%)	13 (13.8%)	65 (22.3%)	0.074	2 ( 7.4%)	76 (21.2%)	0.085	13 (13.5%)	65 (22.5%)	0.059
No job	24 (6.2%)	13 (13.8%)	11 ( 3.8%)	< 0.001	1 ( 3.7%)	23 ( 6.4%)	0.570	13 (13.5%)	11 ( 3.8%)	< 0.001
Student	7 (1.8%)	0 ( 0.0%)	7 ( 2.4%)	0.130	0 ( 0.0%)	7 ( 2.0%)	0.460	0 ( 0.0%)	7 ( 2.4%)	0.120
Others	3 (0.8%)	1 ( 1.1%)	2 ( 0.7%)	0.720	0 ( 0.0%)	3 ( 0.8%)	0.630	1 ( 1.0%)	2 ( 0.7%)	0.740

The numbers in the table represent N (%) for discrete variables and mean (SD) for continuous variables. *P*-values represent the *p*-value resulting from the Pearson's chi-square test for discrete variables and the t-test for continuous variables

*HMB* Heavy menstrual bleeding, *IV* Intravenous, *SD* Standard deviation

^a^Patients who are visiting a gynecologist for any symptoms

^b^Only patients who are on menstruating period can respond

^c^Only patients who have nausea can respond (N = 94)

^d^Only patients who have vomiting can respond (N = 27)

The mean ages (± SD) of the patients with nausea, vomiting, and nausea/vomiting were 39.6 ± 8.0, 38.5 ± 6.8, and 39.6 ± 8.0, respectively, which were significantly lower than those of patients without each of these symptoms (42.3 ± 7.5, *p* = 0.004, 41.9 ± 7.8, *p* = 0.030, and 42.3 ± 7.5, *p* = 0.002, respectively). In terms of primary disease, patients with nausea, vomiting, and nausea/vomiting had a significantly higher proportions of having dysmenorrhea (36.2% vs. 18.6%, *p* < 0.001, 55.6% vs. 20.4%, *p* < 0.001, and 37.5% vs. 18.0%, *p* < 0.001) and PMS (30.9% vs. 11.7%, *p* < 0.001, 51.9% vs. 13.7%, *p* < 0.001, and 32.3% vs. 11.1%, *p* < 0.001) compared to those without each of these symptoms. Regarding medication use, a higher proportion of patients with nausea, with vomiting, and with nausea/vomiting used iron (intravenous) than those without each of these symptoms (16.0% vs. 8.2%, *p* = 0.031, 29.6% vs. 8.7%, *p* < 0.001, and 15.6% vs. 8.3%, *p* = 0.039).

Among all population, 83.4% had symptoms of anemia at the time of the survey; the mean (± SD) duration of symptoms among the patients with nausea was 17.0 ± 101.7 days, with a NRS score of 4.5 ± 2.2. The mean duration of symptoms in patients with vomiting was 40.4 ± 189.8 days and the mean frequency of vomiting per day was 2.0 ± 2.2 times. Note that the duration of each of the symptoms of nausea and vomiting included one case who responded 990 days for each.

### QOL

**Fig. 2:**
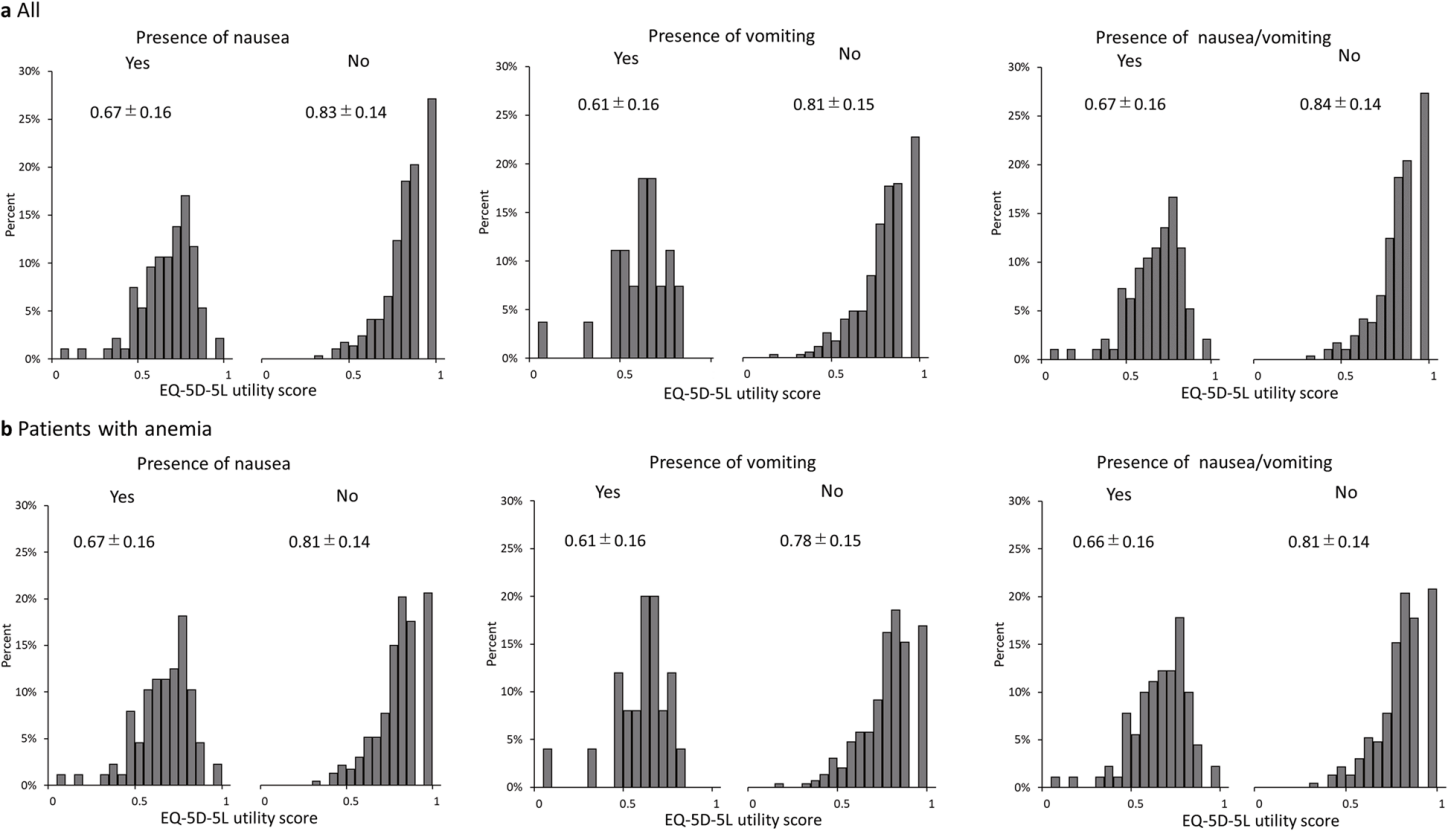
Histograms of EQ-5D-5L utility score. **a** All (N = 385), **b** Patients with anemia (N = 321), Mean ± Standard Deviation

Descriptive statics for all patients and patients with the presence of nausea or vomiting with the EQ-5D-5L utility score and EQ VAS score are summarized in Table 2. The EQ-5D-5L utility score (Mean ± SD) of all the participants was 0.79 ± 0.16. The EQ-5D-5L utility scores for patients with nausea, vomiting, and nausea/vomiting were 0.67 ± 0.16, 0.61 ± 0.16, and 0.67 ± 0.16, respectively, which were significantly lower than those of patients without each of these symptoms (0.83 ± 0.14, 0.81 ± 0.15, and 0.84 ± 0.14, *p* < 0.001 for each). The percentage of respondents in full health with an EQ-5D-5L utility score of 1 was more than 20% in each group of patients without nausea or vomiting, but it tended to be lower in patients with nausea, vomiting, and nausea/vomiting, at 2.1%, 0%, and 2.1%, respectively (Fig. 2a).

**Table 2:** Health outcomes by presence of nausea or vomiting

	All	Presence of nausea			Presence of vomiting			Presence of nausea/vomiting		
	Total	Yes	No	*p-*value	Yes	No	*p-*value	Yes	No	*p-*value
All population										
N	385	94	291	-	27	358	-	96	289	-
EQ-5D-5L										
Utility score	0.79 (0.16)	0.67 (0.16)	0.83 (0.14)	<0.001	0.61 (0.16)	0.81 (0.15)	<0.001	0.67 (0.16)	0.84 (0.14)	<0.001
EQ-5D VAS										
VAS score	59.9 (24.4)	43.6 (21.7)	65.1 (22.9)	<0.001	38.1 (22.9)	61.5 (23.7)	<0.001	43.5 (21.7)	65.3 (22.8)	<0.001
WPAI										
OWI	37.6% (30.3%)	53.0% (26.6%)	32.6% (29.8%)	<0.001	62.4% (22.2%)	35.2% (30.0%)	<0.001	53.5% (26.4%)	32.2% (29.8%)	<0.001
Absenteeism	6.4% (15.9%)	9.8% (15.7%)	5.3% (15.8%)	0.050	10.2% (15.9%)	6.0% (15.9%)	0.230	9.5% (15.5%)	5.3% (15.9%)	0.067
Presenteeism	35.6% (29.2%)	49.5% (25.0%)	31.0% (29.0%)	<0.001	59.1% (22.1%)	33.3% (28.8%)	<0.001	50.2% (24.9%)	30.6% (28.9%)	<0.001
AI	41.0% (29.5%)	54.0% (25.2%)	36.8% (29.7%)	<0.001	60.7% (23.7%)	39.5% (29.4%)	<0.001	54.3% (25.0%)	36.6% (29.7%)	<0.001
Patients with anemia										
N	321	88	233	-	25	296	-	90	231	-
EQ-5D-5L										
Utility score	0.77 (0.16)	0.67 (0.16)	0.81 (0.14)	<0.001	0.61 (0.16)	0.78 (0.15)	<0.001	0.66 (0.16)	0.81 (0.14)	<0.001
WPAI										
OWI	39.6% (30.0%)	53.9% (26.4%)	34.1% (29.6%)	<0.001	62.3% (22.7%)	37.1% (29.7%)	<0.001	54.4% (26.1%)	33.6% (29.5%)	<0.001
Absenteeism	6.9% (16.6%)	9.9% (16.0%)	5.8% (16.7%)	0.100	10.0% (16.2%)	6.6% (16.6%)	0.360	9.6% (15.8%)	5.9% (16.8%)	0.130
Presenteeism	37.3% (28.9%)	50.5% (24.8%)	32.2% (28.8%)	<0.001	59.1% (22.7%)	34.9% (28.5%)	<0.001	51.1% (24.6%)	31.7% (28.7%)	<0.001
AI	44.1% (28.9%)	55.3% (25.2%)	39.9% (29.2%)	<0.001	61.6% (24.4%)	42.7% (28.8%)	0.002	55.6% (25.0%)	39.7% (29.2%)	<0.001

The numbers in the table represent mean (SD). *P*-values represent the *p*-value resulting from the t-test

*EQ-5D-5L* 5-level EQ-5D version, *WPAI* Work Productivity and Activity Impairment, *OWI* Overall Work Impairment, *AI* Activity Impairment, *SD* Standard deviation

In a sensitivity analysis of 321 patients with anemia, the EQ-5D-5L utility score (Mean ± SD) for patients with nausea, vomiting, and nausea/vomiting was 0.67 ± 0.16, 0.61 ± 0.16, and 0.66 ± 0.16, respectively, similar to those of all population (Table 2 and Fig. 2b).

**Fig. 3:**
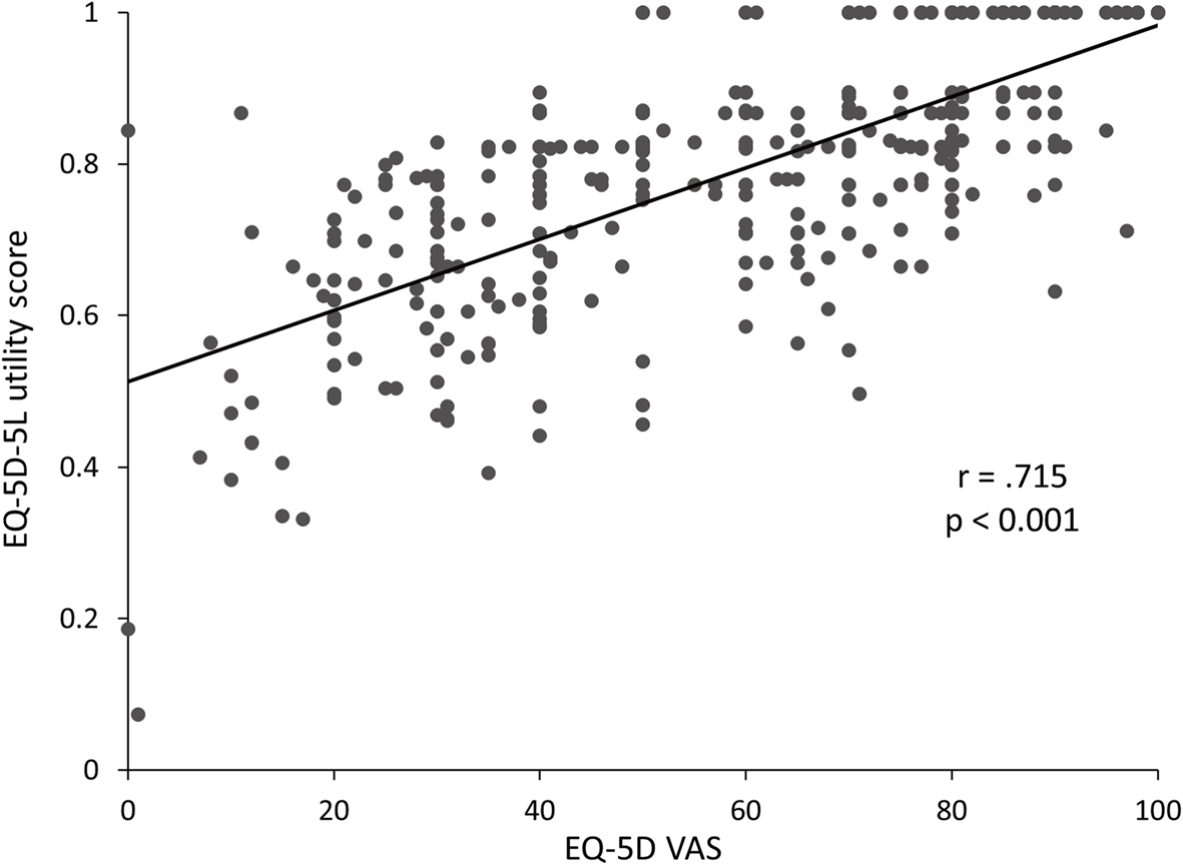
Correlation between EQ-5D-5L utility score and EQ-5D VAS score (N = 385). VAS visual analogue scale

**Fig. 4:**
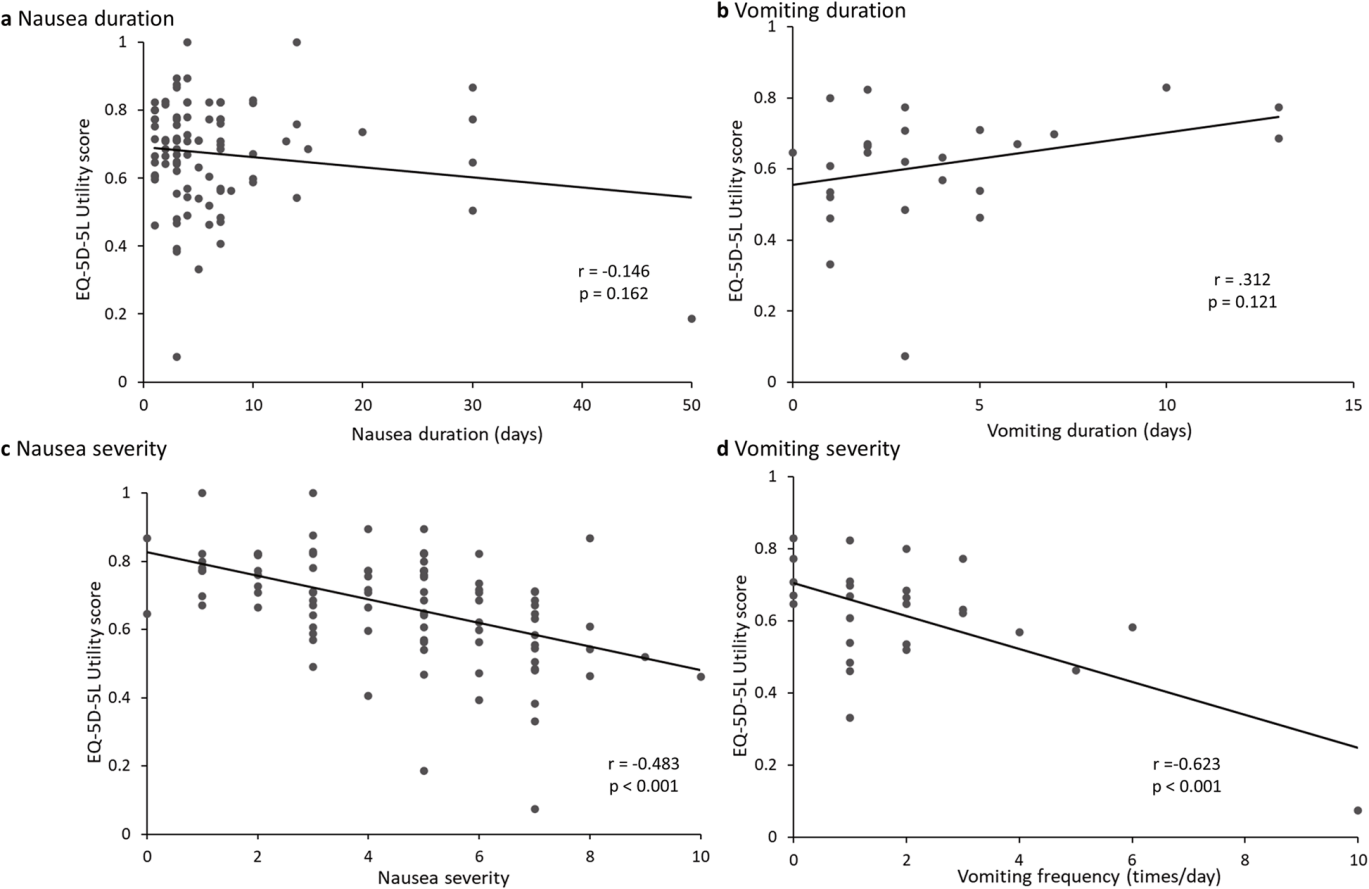
Correlation between health utility scores and duration of symptom/severity. **a** Nausea duration (N = 93), **b** Vomiting duration (N = 26),** c** Nausea severity (N = 93), **d** Vomiting severity (N = 26). One patient in which duration of nausea and vomiting was extremely long, 990 days, was excepted

The association between the EQ VAS score and the EQ-5D-5L utility score was evaluated and a correlation was found between both scores (*r* = 0.715, *p* < 0.001) (Fig. 3). The relationships of duration and severity of nausea, duration of vomiting, and frequency of vomiting to the respective EQ-5D-5L utility scores are shown in Fig. 4. The analysis was performed for each case except one in which the duration of nausea and vomiting was extremely long, 990 days. The correlation coefficients of the EQ-5D-5L utility score with the duration of nausea and the duration of vomiting were -0.146 and 0.312, respectively, showing no association (*p* = 0.162, 0.121, respectively) (Fig. 4a, b). The correlation coefficient of the EQ-5D-5L utility score with the severity of nausea and the frequency of vomiting per day was -0.483 and -0.623, respectively (*p* < 0.001 for each). Decrement QOL was associated with the severity of nausea and the frequency of vomiting (Fig. 4c, d).

### Work productivity impairment

**Fig. 5:**
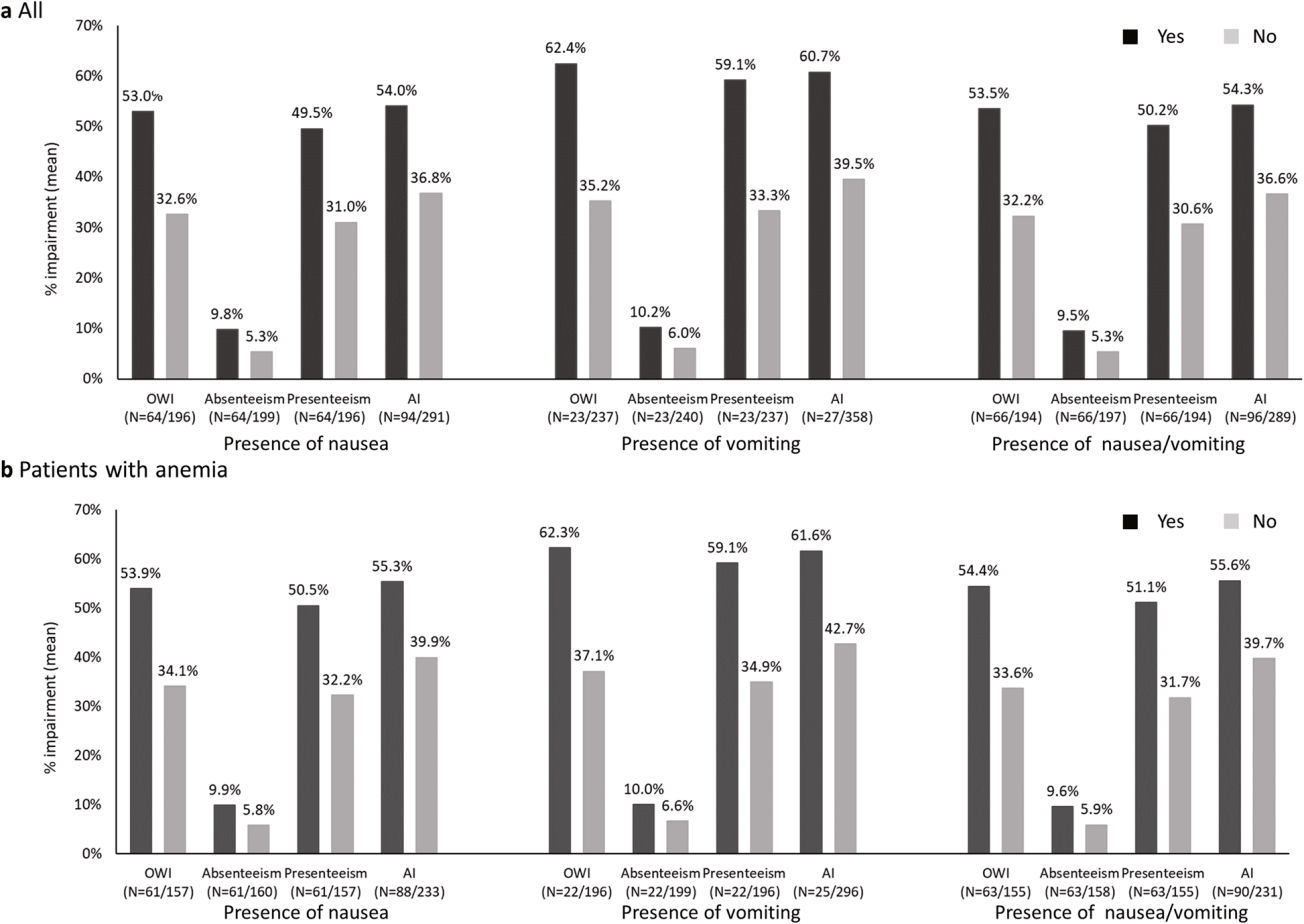
Comparison of work productivity and activity impairment (WPAI) in patients with and without nausea or vomiting. **a** All (N=385), **b** Patients with anemia (N=321), WPAI Work Productivity and Activity Impairment, OWI Overall Work Impairment, AI Activity Impairment

The WPAI scores are shown in Table 2 and Fig. 5. The mean ± SD in OWI of all population was 37.6% ± 30.3%, with the mean absenteeism, presenteeism, and AI of 6.4% ± 15.9%, 35.6% ± 29.2%, and 41.0% ± 29.5%, respectively.

In patients with nausea, vomiting, and nausea/vomiting, the mean OWI was significantly higher compared to those without each of these symptoms (53.0% vs 32.6%, 62.4% vs 35.2% and 53.5% vs 32.2%, *p* < 0.001 for each) and the mean presenteeism was also significantly higher (49.5% vs 31.0%, 59.1% vs 33.3% and 50.2% vs 30.6%, *p* < 0.001 for each). The mean absenteeism tended to be higher in patients with nausea or vomiting, but the difference was not significant. (9.8% vs 5.3%, 10.2% vs 6.0% and 9.5% vs 5.3%, respectively). The mean AI was significantly higher in patients with nausea, vomiting, and nausea/vomiting compared to those without each of these symptoms (54.0% vs 36.8%, 60.7% vs 39.5% and 54.3% vs 36.6%, *p* < 0.001 for each).

In a sensitivity analysis of patients with anemia, as in all population, patients with nausea, vomiting, and nausea/vomiting had significantly higher OWI (*p* < 0.001 for each), presenteeism (*p* < 0.001 for each), and AI (*p* < 0.001, 0.002, < 0.001, respectively) than those without each of these symptoms, and the absenteeism tended to be higher, although no significant difference was observed.

## Discussion

In this study, a cross-sectional web-based survey was conducted in 385 patients taking iron preparations for HMB or anemia, to examine the impacts of nausea and vomiting on QOL and work productivity impairment. As a result, patients with nausea, vomiting, or nausea/vomiting had significantly lower EQ-5D-5L utility scores and significantly higher work productivity impairments (OWI, presenteeism, and AI) than those without such symptoms. To our knowledge, this is the first to examine the impacts of nausea and vomiting on QOL and productivity loss in patients taking iron preparations for HMB or anemia.

As shown in the results, patients with nausea, vomiting, or nausea/vomiting had significantly lower EQ-5D-5L utility scores than those without such symptoms, suggesting that nausea and vomiting are associated with lower QOL. A comparison between patients with vomiting and those with nausea demonstrated that the former had lower mean EQ-5D-5L utility scores than the latter (0.61 vs. 0.67). Patients with nausea included those with vomiting (26.6%) but mostly have only nausea. On the other hand, 92.6% of patients with vomiting also had nausea, suggesting that those with vomiting had both vomiting and nausea. Therefore, patients with vomiting had lower QOL than those with nausea. A study that examined hyperemesis in pregnant women demonstrated that nausea and vomiting lowered QOL [18]. It was shown that the physical and mental component summary scores of Short-Form (SF) 12 were lower in patients with nausea and vomiting than in those only with nausea. This is consistent with the finding that the EQ-5D-5L utility scores of the target population in this study, i.e., patients with vomiting caused by iron preparation intake, were lower than those of patients with nausea [18].

The relationship between the EQ-5D-5L utility and EQ VAS scores was examined, demonstrating a correlation between these scores (*r* = 0.715, *p* < 0.001). The resulting EQ-5D-5L utility scores were consistent with the individual’s rating of overall current health in patients taking iron preparations for HMB or anemia.

The relationship between the EQ-5D-5L utility scores and the duration and severity of nausea/the duration and frequency of vomiting was examined, demonstrating that the EQ-5D-5L utility scores were correlated with the severity of nausea and the frequency of vomiting. Specifically, nausea and vomiting had more significant impacts on QOL as they became severer. On the other hand, the duration of nausea and vomiting was not correlated with the EQ-5D-5L utility scores. This may be explained by the fact that the duration of nausea and vomiting was hardly reflected in the EQ-5D-5L utility scores because the EQ-5D-5L is a questionnaire to determine the current statuses.

In this study, currently employed patients with nausea/vomiting reported a significantly higher impairment of work productivity compared to those without. The mean OWI and presenteeism in patients with nausea/vomiting were 53.5% and 50.2%, respectively, 1.7 and 1.6 times higher than those without nausea/vomiting. Furthermore, patients with nausea/vomiting had a mean AI of 54.3%, 1.5 times higher than those without such symptoms. A previous study that examined the impact of nausea and vomiting, caused by hyperemesis in pregnant women, demonstrated productivity loss [8]. In a study of migraine patients with nausea and vomiting, triptan (most commonly prescribed for the acute treatment of migraine) insufficient responders (TIRs) had a lower mean EQ-5D-5L utility score than triptan responders (TRs) (0.84 vs. 0.91; *p* = 0.001) and impaired work productivity and activity (mean absenteeism, 8.6% vs. 5.1% for TIRs vs. TRs; mean presenteeism, 34.3% vs. 21.0%; mean work impairment, 37.1% vs. 23.3%; mean overall activity impairment, 39.8% vs. 25.3%; all *p* < 0.05) [19]. This study, which examined nausea and vomiting caused by iron preparations, demonstrated that nausea and vomiting have a significant impact on patients' work and daily life, as previously reported. On the other hand, there was no difference in absenteeism (*p* = 0.130) probably because less severe absenteeism does not lead to sick leave and nausea and vomiting caused by iron preparations seldom lead to sick leave.

According to the “Data Health and Collaborative Health Guidelines for Promotion of Corporate Wellness” issued by the MHLW, the overall health-related costs in Japanese employees show the largest percentage for presenteeism (77.9%) but not for medical expenses (15.7%), suggesting that the overall health-related costs, including presenteeism, as well as medical expenses, should be considered for health management [20]. Since nausea and vomiting caused by iron preparations was demonstrated to affect presenteeism in this study, specific productivity loss should be calculated in the future to demonstrate the degree of social impact [21].

This study was conducted in patients taking iron preparations for HMB or anemia. Patients with symptoms of anemia accounted for 83.4% (*n* = 321), suggesting the inclusion of those without anemia symptoms. In the sensitivity analysis to verify the definition of anemia by PRO, the EQ-5D-5L utility and WPAI scores were compared depending on the presence or absence of nausea, vomiting, and nausea/vomiting in patients with symptoms of anemia, yielding almost the same results as for all population. Thus, anemia was considered to be properly defined in this study.

This study was limited as below. Firstly, the study is a cross-sectional design, which precludes the ability to make definitive causal inferences between anemia, nausea, vomiting and the outcomes examined. Secondly, since the survey is a web-survey in patients enrolled in a patient panel, those who did not have access to the Internet, those who were unfamiliar with online surveys, and those who were in poor health were not included, suggesting the insufficient representativeness of patients. However, iron deficiency anemia is common in females in their 30s to 40s [22], suggesting that the mean age (41.6 years) of the patients included should ensure the representativeness to some extent. Thirdly, this study is based on self-reported medication use and current symptoms, suggesting a possible discrepancy with physicians’ evaluations and laboratory values. However, incorrect responses were prevented as much as possible through the wording of questions and addition of explanations. Fourthly, educational backgrounds of patients were not investigated. Fifthly, because this study was designed to be descriptive research, we did not conduct the comparative analysis after adjusting for factors such as age that may affect QOL and impairment of work productivity. Finally, although this study provided descriptive statistics on the relationship between nausea/vomiting and impairment of work productivity, the impacts of factors other than nausea and vomiting, which may influence the impairment of work productivity, could not be examined.

## Conclusions

This study showed that patients taking iron preparations for HMB or anemia with nausea or vomiting experience a significant burden in terms of poorer QOL and higher work productivity impairment. These results indicate that there is an unmet need for better interventions and treatments to improve iron deficiency anemia without causing nausea and vomiting.

## Data Availability

The datasets generated and/or analyzed during the study are available from the corresponding author on reasonable request.
